# Robotic-assisted combined transposition of left renal vein and gonadal vein as a novel treatment option for renal nutcracker syndrome: A case report

**DOI:** 10.1097/MD.0000000000032509

**Published:** 2023-01-13

**Authors:** Hua Wang, Zhansen Huang, Cheng Hu, Jieying Wu, Qunxiong Huang, Tengcheng Li, Jinming Di

**Affiliations:** a Department of Urology, The Third Affiliated Hospital, Sun Yat-Sen University, Guangzhou, Guangdong, China.

**Keywords:** combined veins transposition, nutcracker syndrome, robotic surgery

## Abstract

Renal nutcracker syndrome is a rare phenomenon that often causes various disability symptoms. The treatment protocol has been explored for a long time, but no consensus has been reached.

**Patient concerns:**

Here, we report the case of a 19-year-old male suffering with nutcracker syndrome, including left-sided flank pain and intermittent gross hematuria.

**Diagnoses:**

The patient was diagnosed with renal nutcracker syndrome, and the pressure gradient between the left renal vein and inferior vena cava was >5 mm Hg.

**Interventions:**

The patient underwentrobotic-assisted combined transposition of left renal vein and gonadal vein.

**Outcomes:**

Flank pain and gross hematuria ceased spontaneously after surgery without occurrence.

**Lessons:**

Robotic-assisted combined transposition of the left renal vein and gonadal vein is a safe and promising option for this condition.

## 1. Introduction

Renal nutcracker syndrome (RNS) refers to a series of clinical symptoms causes by left renal vein (LRV) compression, including flank pain, hematuria, orthostatic proteinuria and fatigue.^[1]^ Patients have to ultimately receive surgical management while conservative treatment is not working. Surgical strategy is various which can be roughly divided into 2 classes, transposition or bypass of the vein and endovascular therapy.^[2]^ A consensus on the stander treatment of this rare syndrome has not been reached because of varying degrees of success. Herein, we firstly reported a case of RNS with severe left-side flank pain, gross hematuria and proteinuria, who was cured after Robotic-assisted laparoscopic combined transposition of LRV and gonadal vein.

## 2. Case report

This study was approved by the Medical Ethics Committee of Sun Yat-Sen University, and informed consent was obtained from the patient forpublication of this case.

**Figure 1.**
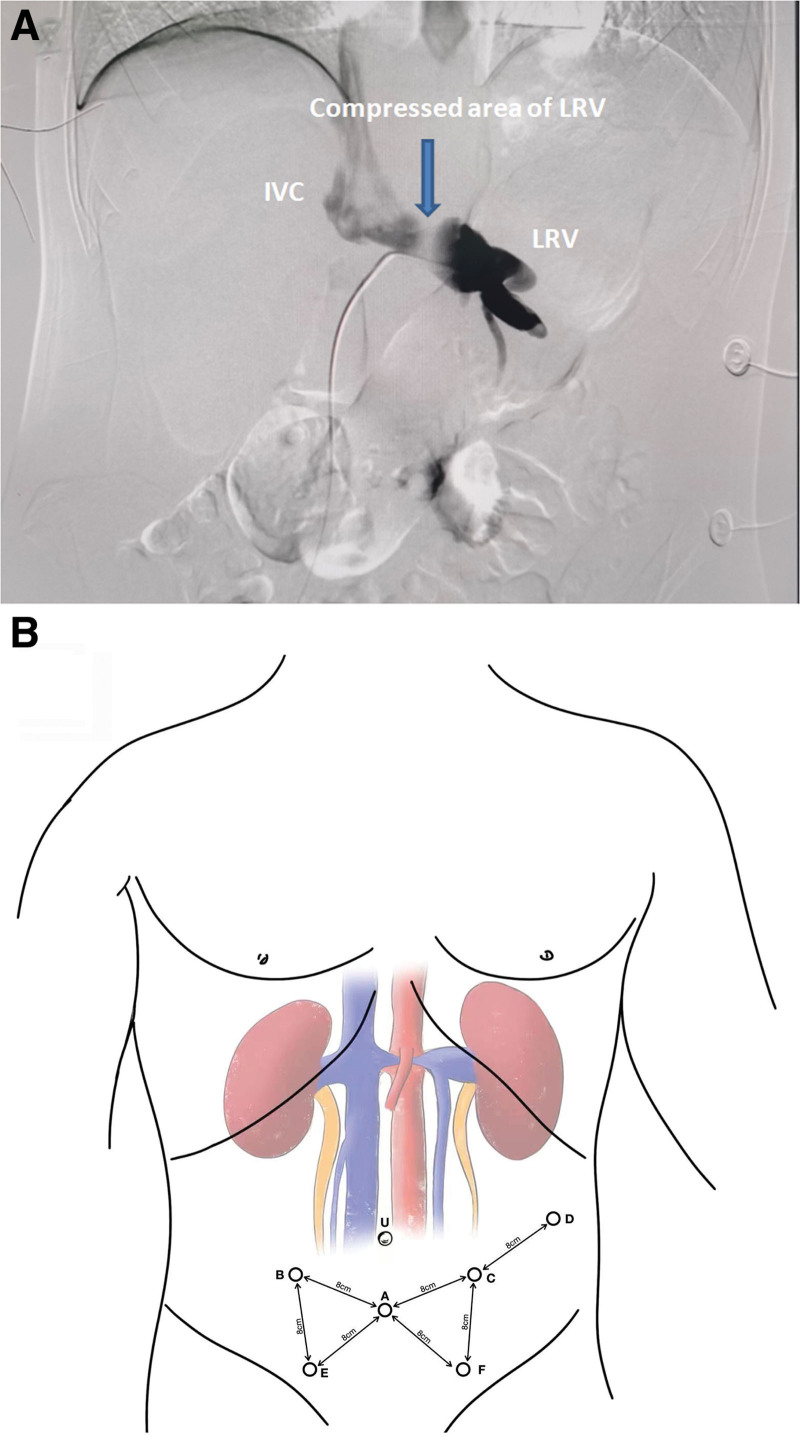
(A) Venogram showed filling defect in left renal vein (LRV) and pressure gradient was >5 mm Hg. (B) Overview of the location of all ports. (A) represent Camera port; (B–D) represent 3 robotic arms; (E–F) represent the assistant ports; U = umbilicum.

A 19-year-old male complained left-sided flank pain and intermittent gross hematuria. Urinalysis revealed hematuria and proteinuria whereas without any infectious evidence. Furthermore, the patient underwent a follow-up enhanced CT imaging revealed that LRV compression and angle between aorta and superior mesenteric artery (SMA) was 14 degrees. Renal venography furthermore confirmed vein compression, we observed the pressure gradient between the LRV and inferior vena cava (IVC) was >5 mm Hg (Fig. 1A). Moreover, all examinations or tests excluded the possibility of other diseases, such as nephrosis, urolithiasis and tumor.

**Figure 2.**
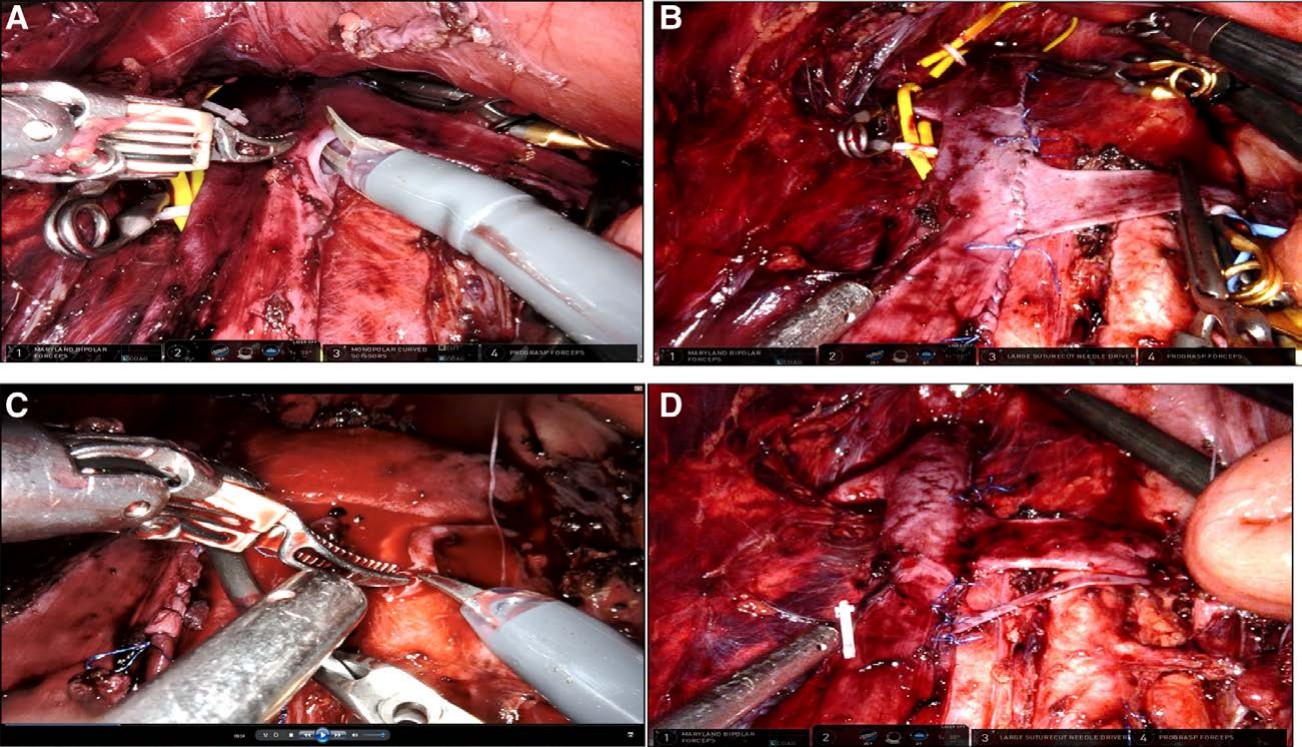
(A) LRV was transected from IVC. (B) LRV was reanastomosed on the IVC approximately 5 cm more distally. (C) Left gonadal vein was transected. (D) Proximal gonadal vein was distally anastomosed on the IVC. LRV = left renal vein, IVC = inferior vena cava.

**Figure 3.**
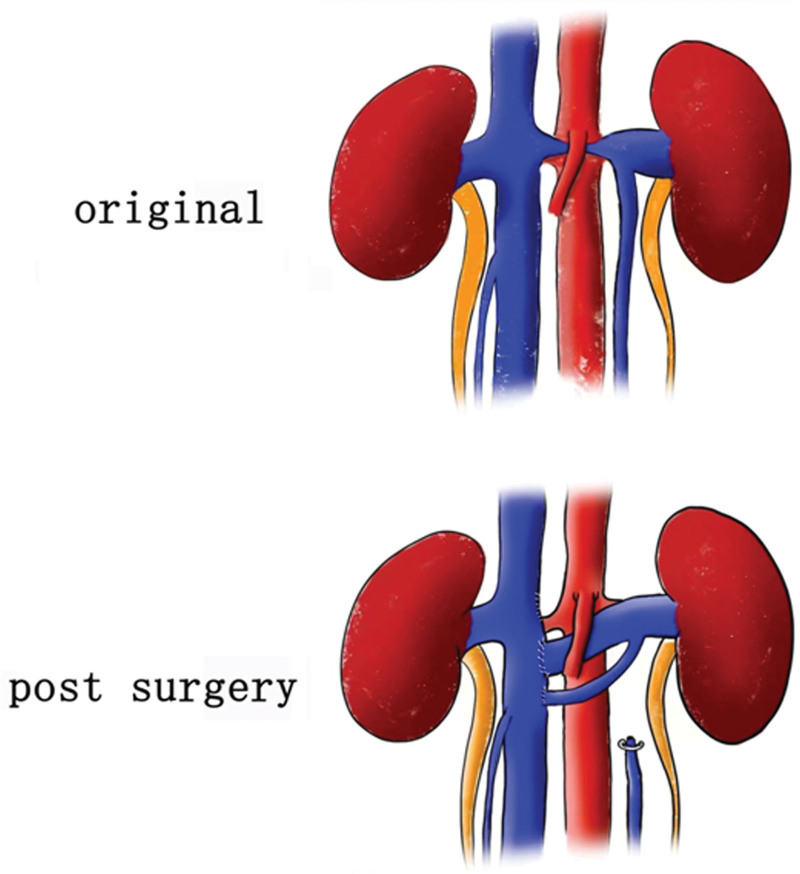
Illustration shows the changing of regional vascular anatomy.

Under general anesthesia, the patient was placed in trendelenburg position. Standard sterile preparation and draping were performed. Two 12-mm ports and 3 8-mm ports were used. All ports were placed similar to Lacy Harkrader’s report(Fig. 1B).^[3]^ The robotic system (Da Vinci Robot XI) was docked and connected to the ports. Procedures were mainly carried out as follows: 1. Peritoneum of the mesenteric root was opened in a fan shape by using Hem-o-lok clip-with-line. 2. The aorta and IVC were identified superiorly and then IVC was mobilized circumferentially until bilateral renal veins appeared. Then, bilateral renal veins were mobilized and all branches of LRV were ligated by using Hem-o-lok clips. 3. Vessel Loops were used to control suprarenal IVC, infrarenal IVC, and right renal vein and bulldog clamp complete occluded these vessels. 4. The LRV was transected and then reanastomosed laterally on the IVC approximately 5 cm more distally with a running 5 to 0 Prolene suture (Fig. 2A and B). The proximal gonadal vein was astomosed to IVC and procedures were similar to transposition of LRV (Fig. 2C and D). The final anatomy of regional vascular was illustrated in Figure 3. Total operation time was 6 hours and the time of renal ischemia was 30 minutes. The estimated blood loss was 50 mL. During postoperative days, intravenous antibiotic was given and heparin was avoided. Gross hematuria and proteinuria were disappeared 3 days after surgery. And the flank pain ceased spontaneously 2 months after surgery without recurrence. The symptom of microhematuria was gradually improved at 6 months follow-up while there was not a relapse of other symptoms.

## 3. Discussion

RNS is also known as LRV entrapment. It often manifests as hematuria, orthostatic proteinuria, low back and abdominal pain and varicocele. This rare phenomenon was firstly reported by Grant1 in 1944.^[4]^ However, RNS has raised widespread concern until Schepper firstly described nephrorrhagia caused by this disease in 1972.^[5]^

Ultrasonography is the first choice in diagnosis of RNS. Respectively, the sensitivity, specificity, and accuracy of Ultrasonography are 80%, 94%, and 83%.^[6]^ CT and MR can not only accurately measure the angle between the SMA and the aorta but also clearly show the compression of the LRV and the expansion of the gonadal vein, which are ideal noninvasive examinations. However, the current gold standard examination for the diagnosis of RNS are renal venography and intravascular ultrasound but both of them are invasive.^[7]^ RNS can be established while the pressure gradient between the LRV and the IVC is >3 mm Hg.^[8]^ In our center, the patient’s CT showed that the angle between the abdominal aorta and the SMA was 14 degrees. Then, the renal venography demonstrated that pressure gradient between the LRV and the IVC was >5 mm Hg, which confirmed the diagnosis of RNS.

Usually, teenager present with mild hematuria or proteinuria are given priority to observe for about 2 years.^[9]^ Patient suffer with severe flank pain, repeated gross hematuria and renal function damage always need to undergo surgical intervention. Although renal venous bypass, autotransplantation of the kidney and endovascular stent implantation had been reported, there was still no unified treatment plan for the rare symptom.

Markus et al firstly reported the long term follow-up result of 8 patients underwent transposition of the LRV for treatment of the RNS.^[10]^ This study has established the treatment efficiency of opened transposition of the LRV. Then, Wang et al reported a 26-year-old man complained hematuria who was the first case cure by robotic-assisted laparoscopic transposition of the LRV.^[11]^ Different from traditional procedures which routinely dissected and ligated gonadal vein during transposition of the LRV, we tentatively anastomosed proximal gonadal vein into the IVC in a 19-year-old male patient. Since left gonadal vein of patient suffer with RNS always dilated, we speculate this manipulation may not only capable of carrying a sufficient amount of renal venous outflow to decompress the kidney but also decreasing renal vein pressure, thus help to relieve the flank pain and hematuria. Similar research has supported our speculation.^[12]^ In addition to avoiding heparin use after surgery, we believed the management may also effectively decrease the risk of thrombosis or stenosis of LRV. As far as we know, this was the first report of combined transposition of the LRV and gonadal vein to cure RNS by robotic approach. Due to insufficient number of cases, however, superiority of the technique should be further confirmed.

## 4. Conclusion

In this report, the patient’s symptoms improved significantly after receiving Robotic-assisted combined transposition of left renal vein and gonadal vein. This technique was not only provided a new choice of treatment but also a novel therapeutic concept for RNS. Of course, long-term recovery of the patient needs further follow-up.

## Author contributions

**Writing – original draft:**Hua Wang, Zhansen Huang.

**Writing – review & editing:** Jinming Di, Cheng Hu, Jieying Wu, Qunxiong Huang, Tengcheng Li.
